# Using X-ray Crystallography, Biophysics, and Functional Assays to Determine the Mechanisms Governing T-cell Receptor Recognition of Cancer Antigens

**DOI:** 10.3791/54991

**Published:** 2017-02-06

**Authors:** Bruce J. MacLachlan, Alexander Greenshields-Watson, Georgina H Mason, Andrea J Schauenburg, Valentina Bianchi, Pierre J Rizkallah, Andrew K Sewell, Anna Fuller, David K Cole

**Affiliations:** ^1^Division of Infection and Immunity and Systems Immunity Research Institute, Cardiff University; ^2^Department of Oncology, University Hospital of Lausanne (CHUV); ^3^Ludwig Insitutue for Cancer Research, Lausanne Branch, University of Lausanne

**Keywords:** Immunology, Issue 120, CD8+ T cells, T-cell receptor (TCR), peptide-human leukocyte antigen (pHLA), surface plasmon resonance, X-ray crystallography, cancer, gp100, melanoma, heteroclitic peptides

## Abstract

Human CD8+ cytotoxic T lymphocytes (CTLs) are known to play an important role in tumor control. In order to carry out this function, the cell surface-expressed T-cell receptor (TCR) must functionally recognize human leukocyte antigen (HLA)-restricted tumor-derived peptides (pHLA). However, we and others have shown that most TCRs bind sub-optimally to tumor antigens. Uncovering the molecular mechanisms that define this poor recognition could aid in the development of new targeted therapies that circumnavigate these shortcomings. Indeed, present therapies that lack this molecular understanding have not been universally effective. Here, we describe methods that we commonly employ in the laboratory to determine how the nature of the interaction between TCRs and pHLA governs T-cell functionality. These methods include the generation of soluble TCRs and pHLA and the use of these reagents for X-ray crystallography, biophysical analysis, and antigen-specific T-cell staining with pHLA multimers. Using these approaches and guided by structural analysis, it is possible to modify the interaction between TCRs and pHLA and to then test how these modifications impact T-cell antigen recognition. These findings have already helped to clarify the mechanism of T-cell recognition of a number of cancer antigens and could direct the development of altered peptides and modified TCRs for new cancer therapies.

**Figure:**
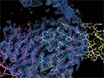


## Introduction

X-ray crystallography has been, and will continue to be, an extremely powerful technique to understand the nature of ligand-receptor interactions. By visualizing these interactions in atomic detail, not only is it possible to divulge the molecular mechanisms governing many biological processes, but it is also possible to directly alter contact interfaces for therapeutic benefit. Coupled with techniques such as surface plasmon resonance and isothermal titration calorimetry (to name just a couple), such modifications can then be analyzed biophysically to assess the direct impact on binding affinity, interaction kinetics, and thermodynamics. Finally, by performing functional experiments on relevant cell types, a detailed picture of the molecular and functional impact of modifications to receptor-ligand interactions can be gleaned, providing very specific mechanistic information. Overall, these types of methods provide an atomic resolution picture enabling the determination of how biological systems work, with attendant implications for diagnostic and therapeutic advances.

Our laboratory routinely uses these techniques to study the receptors that mediate human T-cell immunity to pathogens and cancer, in autoimmunity, and during transplantation. Here, we focus on the human CD8^+^ T-cell response to cancer, mediated by an interaction between the T-cell receptor (TCR) and human leukocyte antigen (HLA)-restricted tumor-derived peptides (pHLA). This is important because, although CD8^+^ T cells are able to target cancer cells, we and others have previously shown that anti-cancer TCRs suboptimally bind to their cognate pHLA^1^,^2^. Thus, many laboratories have attempted to alter either the TCR^3^,^4^,^5^ or the peptide ligand^6^,^7^,^8^ in order to increase immunogenicity and to better target cancer cells. However, these approaches are not always effective and can have severe side effects, including off-target toxicities^4^,^9^,^10^. Further research exploring the molecular mechanisms that govern T-cell recognition of cancer antigens will be vital to overcome these shortfalls.

In the present study, we focused on the responses against autologous melanoma cells by CD8^+^ T cells specific for a fragment of the differentiation melanocyte antigen glycoprotein 100 (gp100), gp100_280-288_, presented by HLA-A*0201 (the most commonly-expressed human pHLA class I). This antigen has been a widely studied target for melanoma immunotherapy and has been developed as a so-called "heteroclitic" peptide in which a valine replaces alanine at anchor position 9 to improve pHLA stability^11^. This approach was used to enhance the induction of melanoma-reactive CTLs *in vitro* and has been successfully used in clinical trials^12^. However, modifications to peptide residues can have unpredictable effects on T-cell specificity, demonstrated by the poor efficacy of most heterolitic peptides in the clinic^6^,^13^. Indeed, another heteroclitic form of gp100_280-288_, in which peptide residue Glu3 was substituted for Ala, abrogated recognition by a polyclonal population of gp100_280-288_-specific T cells^14^,^15^. We have previously demonstrated that even minor changes in peptide anchor residues can substantially alter T-cell recognition in unpredictable ways^6^,^16^. Thus, the study focused on building a more detailed picture of how CD8^+^ T cells recognize gp100 and how modifications of the interaction between TCRs and pHLA could impact this function.

Here, we generated highly pure, soluble forms of two TCRs specific for gp100_280-288_ presented by HLA-A*0201 (A2-YLE), as well as the natural and altered forms of pHLA. These reagents were used to generate protein crystals to solve the ternary atomic structure of a human TCR in complex with the heteroclitic form of A2-YLE, as well as two of the mutant pHLAs in unligated form. We then used a peptide scanning approach to demonstrate the impact of peptide substitutions on TCRs by performing in-depth biophysical experiments. Finally, we generated a genetically modified CD8+ T-cell line, re-programmed to express one of the A2-YLE-specific TCRs, in order to perform functional experiments to test the biological impact of the various peptide modifications. These data demonstrate that even modifications to peptide residues that are outside of the TCR binding motif can have unpredictable knock-on effects on adjacent peptide residues that abrogate TCR binding and T-cell recognition. Our findings represent the first example of the structural mechanisms underlying T-cell recognition of this important therapeutic target for melanoma.

## Protocol

### 1. Protein Expression

1. Make gene constructs for the generation of soluble TCRs and pHLAs, as described in detail previously^17^,^18^. Design each construct with a 5' BamH1 and a 3' EcoR1 restriction site for insertion into the pGMT7 vector.
2. Transform *E. coli* Rosetta (DE3)pLysS with a pGMT7-derived plasmid vector containing the sequence encoding the protein of interest by incubating 1 µL of 50-200 ng/µL plasmid with 5 µL of *E.coli* for 5 min at 4 °C, 2 min at 42 °C, and 5 min at 4 °C, and plate out overnight at 37 °C on an LB agar plate supplemented with 50 mg/L carbenicillin.
3. Pick individual colonies and grow at 37 °C and 220 rpm in 30 mL of TYP media (16 g/L tryptone, 16 g/L yeast extract, and 5 g/L HK_2_O_4_) supplemented with 100 µM carbenicillin until the suspension reaches an optical density (OD_600_) of between 0.4 and 0.6.
4. Induce protein production in a 5 mL aliquot by introducing 0.5 mM isopropyl β-D-1-thiogalactopyranoside (IPTG) for 3 h. Keep 20 µL of suspension with and without induction for sodium dodecyl sulfate polyacrylamide gel electrophoresis (SDS-PAGE) analysis and stain the gel.
5. Add starter cultures to 1 L of TYP supplemented with 100 µM carbenicillin and grow cells as described above in step 1.3 until the suspension reaches an OD_600_ between 0.4 and 0.6.
6. Induce protein expression for 3 h with 0.5 mM IPTG. Centrifuge the cells for 20 min at 3,000 x g and pour off the supernatant carefully.
7. Dissolve the pellet in 40 mL of lysis buffer (10 mM Tris, pH 8.1; 10 mM magnesium chloride, MgCl_2_; 150 mM NaCl; and 10% glycerol), sonicate on ice for 30 min at 60% power using a 2 s interval, and incubate at room temperature (RT) for 30 min with 0.1 g/L DNase.
8. Treat the suspension containing the proteins in the form of inclusion bodies (IB) with 100 mL of wash buffer (0.5% Triton X-100; 50 mM Tris, pH 8.1; 100 mM NaCl; and 10 mM EDTA).
9. Centrifuge the sample for 20 min at 4 °C and 8,000 x g and pour off the supernatant carefully. Re-suspend the pellet in 100 mL of re-suspension buffer (50 mM Tris, pH 8.1; 100 mM NaCl; and 10 mM EDTA, pH 8.1), centrifuge as before at 8,000 x g, and pour off the supernatant carefully.
10. Finally, dissolve the pellet in 10 mL of guanidine buffer (6 M guanidine; 50 mM Tris, pH 8.1; 2 mM EDTA, pH 8.1; and 100 mM NaCl) and measure the protein concentration at 280 nm using a spectrophotometer.

### 2. pHLA and TCR Refolding

1. For the pHLA refolds, mix 30 mg of HLA-A2 (or HLA-A2 with a biotin tag) IBs, 30 mg of β2m IBs, and 4 mg of peptide for 30 min at 37 °C in a water bath in a final volume of 6 mL of guanidine buffer supplemented with 10 mM dithiothreitol (DTT).
2. Initiate protein refolding by diluting the previous mix in 1 L of a pre-chilled HLA refold buffer (50 mM Tris, pH 8.1; 400 mM L-arginine; 2 mM EDTA, pH 8.1; 6 mM cysteamine; and 4 mM cystamine).
3. Leave the HLA refold stirring at 4 °C for 3 h and then transfer it into a 12.4 kDa MWCO (molecular weight cut-off) dialysis tube and dialyze twice for 24 h against 20 L of 10 mM Tris, pH 8.1.
4. For the TCR refolds, mix 30 mg of TCR α chain IBs and 30 mg of TCR β chain IBs for 30 min at 37 °C in a water bath in 6 mL of guanidine buffer supplemented with 10 mM DTT.
5. Initiate protein refolding by diluting the denatured TCR mixture in 1 L of a pre-chilled TCR refold buffer (50 mM Tris, pH 8.1; 2.5 M urea; 2 mM EDTA, pH 8.1; 6 mM cysteamine; and 4 mM cystamine) for 3 h.
6. Transfer the refold into a 12.4-kDa MWCO dialysis tube and dialyze twice for 24 h against 20 L of 10 mM Tris, pH 8.1.
7. Filter both the pHLA or TCR refolds using a 0.45-µm membrane filter for the purification steps.

### 3. Purification by Fast Protein Liquid Chromatography (FPLC)

1. Load the filtered refold preparation (either pHLA or TCR) onto a 7.9 mL, 50 µm anion exchange resin column pre-equilibrated with 20 mL of 10 mM Tris, pH 8.1 on a flexible and intuitive chromatography system.
2. Elute the protein at 5 mL/min with a salt gradient (0-500 mM NaCl in 10 mM Tris, pH 8.1, over 8 column volumes) and collect 1-mL fractions.
3. Analyze the fractions corresponding to the protein of interest by SDS-PAGE, pool the fractions containing the protein of interest together, and concentrate them down to 500 µL with 10-kDa MWCO 20 or 10 kDa MWCO 4 by centrifugation for 20 min at 4,000 x g, discarding the flow-through.
4. Load the concentrated protein preparations into a 2 mL injection loop onto a 24 mL size exclusion chromatography column pre-equilibrated with the appropriate elution buffer: phosphate-buffered saline (PBS), HBS (10 mM HEPES, pH 7.4; 150 mM NaCl; 3.4 mM EDTA; and 0.005% surfactant), or crystal buffer (10 mM Tris, pH 8.1, and 10 mM NaCl).
5. Elute the proteins at a flow-rate of 0.5 mL/min over 1 column volume; collect 1 mL fractions containing the protein of interest verified by SDS-PAGE. NOTE: These methods were used to generate soluble PMEL17 TCR and gp100 TCRs, as well as all of the pHLAs used in this study: HLA-A*0201 with YLEPGPVTA (A2-YLE), YLEPGPVTV (A2-YLE-9V), ALEPGPVTA (A2-YLE-1A), YLAPGPVTA (A2-YLE-3A), YLEAGPVTA (A2-YLE-4A), YLEPAPVTA (A2-YLE-5A), YLEPGAVTA (A2-YLE-6A), YLEPGPATA (A2-YLE-7A), or YLEPGPVAA (A2-YLE-8A).

### 4. Surface Plasmon Resonance (SPR) Analysis

1. Perform equilibrium-binding analysis or thermodynamic analysis using a molecular interaction analysis system equipped with a CM5 sensor chip^19^.
2. Activate the CM5 chip by flowing a 1:1 mix of 100 mM N-hydroxysuccinimide (NHS) and 400 mM 1-ethyl-3-(3-dimethylpropyl)-carboiimide (EDC) for 10 min at a flow rate of 10 µL/min and at 25 °C.
3. Load approximately 5,000 response units (RU) of streptavidin (110 µL of 200 µg/mL in 10 mM acetate, pH 4.5) by covalent linking to the chip surface in all four flow-cells and use 100 µL of 1 M ethanolamine hydrochloride to deactivate any remaining reactive groups.
4. Couple approximately 500-600 RU of pHLA, at ~1 µM in commercial buffer (provided by the manufacturer), to the CM5 sensor chip at a slow flow-rate of 10 µL/min to ensure uniform distribution on the chip surface.
5. Saturate the chip surface with 1 mM biotin in commercial buffer (provided by the manufacturer) for 60 s.
6. Inject ten serial dilutions of soluble TCRs over the relevant flow-cells at a high flow-rate of 30 µL/min at 25 °C.
7. Calculate the equilibrium-binding constant (K_D _(E)) values using a nonlinear curve fit (y = (P1x)/(P2+x)) 20. NOTE: y = response units, x = analyst concentration, P1 = r_max_, P2 = K_D_.
8. Perform kinetics analysis assuming a 1:1 Langmuir binding and fit the data using a global-fit algorithm in the software package^2^.
9. Perform thermodynamics experiments by repeating this method at the following temperatures: 5 °C, 12 °C, 18 °C, 25 °C, and 30 °C^19^.
10. Use the K_D_ values determined by SPR at different temperatures to calculate ΔG° using the standard thermodynamic equation (ΔG° = -RTlnK_D_)^19^. NOTE: R = gas constant, T = temperature in K, ln = natural log.
11. Calculate the thermodynamic parameters according to the Gibbs-Helmholtz equation (ΔG° = ΔH − TΔS°)^19^.
12. Plot the binding free energies, ΔG° (ΔG° = -RTlnK_D_), against temperature (K) using a nonlinear regression to fit the three-parameter equation (y = ΔH+ΔCp*(x-298)-x*ΔS-x*ΔCp*ln(x/298))^19^. NOTE: y = temperature in K, x = ΔG°.

### 5. Isothermal Titration Calorimetry (ITC)

1. Perform ITC experiments using an isothermal titration calorimeter. Inject 30 µM pHLA into the calorimeter cell and load 210 µM soluble TCR into the syringe. Use the following buffer conditions: 20 mM Hepes (pH 7.4) containing 150 mM NaCl.
2. Perform 20 TCR injections, each of a 2 µL volume. Calculate ΔH and K_D_ using analytical software.

### 6. Crystallization, Diffraction Data Collection, and Model Refinement

1. Perform crystallization trials using a crystallization robot.
2. Grow crystals by vapor diffusion at 18 °C via the sitting drop technique in a 96-well plate with a reservoir containing 60 µL of crystallization buffer (mother liquor)^21^.
3. Concentrate the soluble pHLA to approximately 10 mg/mL (0.2 mM) in crystal buffer by spinning at 3,000 x g in a 10-kda molecular weight cut-off centrifugal concentrator.
4. For the co-complex structures, mix the TCR and pHLA at a 1:1 molar ratio to obtain a protein solution at approximately 10 mg/mL (0.1 mM).
5. Add 200 nL of pHLA alone, or the 1:1 molar ratio mix of TCR and pHLA, to 200 nL of each reservoir solution from the crystallization screen using a crystallization robot and score for crystals under a microscope after 24 h, 48 h, 72 h, and then once a week.
6. Harvest single crystals by manually mounting them in cryo-loops under a microscope and cryo-cool them by submerging and storing them in liquid nitrogen (100 K). NOTE: Loading crystals takes a bit of practice, and deciding which crystals are good enough for data collection comes with experience. As a rule of thumb, the larger and more regular the crystal, the better.
7. Collect data in a stream of nitrogen gas at 100 K. NOTE: This data was acquired at the Diamond Light Source (DLS) national synchrotron science facility in the UK.
8. Analyze the data by estimating the reflection intensities with xia2 using both MOSFLM^22^ and XDS packages^23^, and then scale the data with SCALA or AIMLESS 24 and the CCP4 package^25^.
9. Solve the structures with molecular replacement using PHASER^26^.
10. Adjust the model with COOT^27^ and refine the model with REFMAC5^28^.
11. Prepare graphical representations with PYMOL^29^.
12. Calculate the contacts by using the "contact" program in the CCP4 package. Use a 4 Å cut-off for van der Waals contacts and a 3.4 Å cut-off for hydrogen bonds and salt bridges.
13. Calculate surface complementarity using the "SC" program in the CCP4 package.
14. Calculate the crossing angle of the TCR-pHLA complex, as described^30^. NOTE: For this study, the reflection data and final model coordinates were deposited with the PDB database (PMEL17 TCR-A2-YLE-9V PDB: 5EU6, A2-YLE PDB: 5EU3, A2-YLE-3A PDB: 5EU4, and A2-YLE-5A PDB: 5EU5).

## Representative Results

Using the methods described above, we generated soluble TCR (**Table 1**) and pHLA molecules to conduct in-depth molecular analyses of gp100_280-288_ recognition by CD8^+^ T cells. A modified *E. coli* expression system was used to generate insoluble IBs for each separate chain of both the TCRs (α and β chains) and pHLAs (α chain and β2m). This method has the advantage of being relatively cheap and easy to set up and generates large yields of protein (100-500 mg/L of culture). Also, the insoluble proteins are highly stable if stored at -80 °C. We then used a well-established refolding and purification technique to generate functional, homogeneous, soluble proteins. This method is useful for generating proteins for biophysical, structural, and cellular experiments, as well as reagents that can be used for diagnostics or therapeutics.

Here, we used these proteins to perform alanine scan mutagenesis experiments across the peptide backbone and evaluated TCR binding affinity using surface plasmon resonance (SPR) experiments (**Table 2**). This assay demonstrated which residues in the peptide were most important for TCR binding. High-resolution analyses of binding affinities using this technique are extremely useful for determining biological mechanisms that control protein-protein interactions, as well as for analyzing the binding affinity of therapeutic molecules.

We then crystallized a melanoma-specific soluble TCR (PMEL17 TCR) in complex with a modified tumor-derived pHLA (A2-YLE-9V) to investigate the binding mode at atomic resolution (**Figures 1 **and** 2 **and** Table 3**). These experiments provide direct visualization of the binding interface between two molecules, providing key information about the underlying principles governing the interaction. We further performed a thermodynamic analysis of the interaction using both SPR and ITC, revealing the energetic contributions that enabled binding (**Figures 3**). These analyses were further supported by a high-resolution description of the contact footprint between the two proteins (**Figure 4 **and** Table 4**).

We then solved the structures of unligated pHLA molecules, presenting mutated forms of the peptide, revealing that a molecular switch could explain why certain mutations abrogated TCR binding (**Figures 5**).

Overall, these techniques provided novel data demonstrating the mechanism explaining how T cells recognize a melanoma-derived antigen that is an important target for anti-cancer therapeutics. More broadly, these techniques can be used to investigate virtually any receptor-ligand interaction, uncovering new biological mechanisms that might be targeted for novel therapeutic advances.


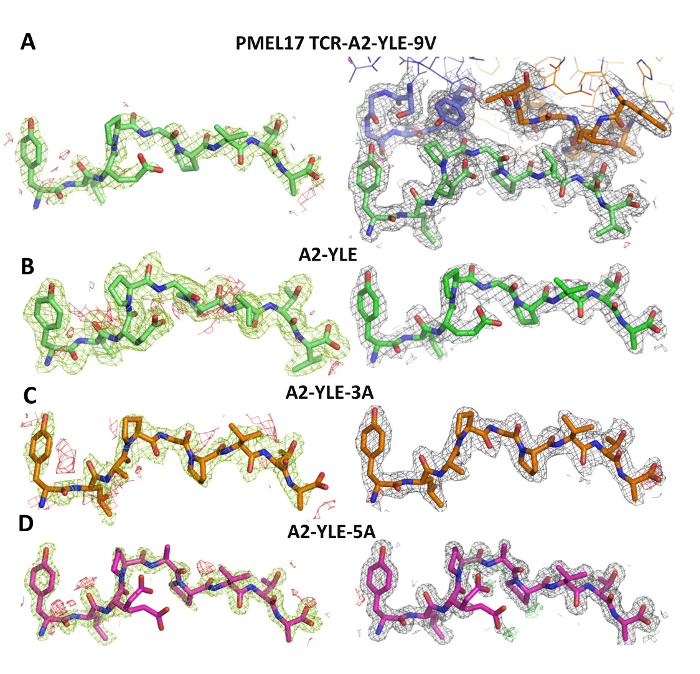
**Figure 1: Density Plot Analysis. **The left column shows omit maps in which the model was refined in the absence of the peptide. Difference density is contoured at 3.0 sigma, positive contours are shown in green, and negative contours are red. The right-hand column shows the observed map at 1.0 sigma (shown as a gray mesh around stick representations of the protein chains) after subsequent refinement using automatic non-crystallographic symmetry restraints applied by REFMAC5. (**A**) The model for PMEL17 TCR-A2-YLE-9V with the TCR CDR3 loops colored blue (α chain) and orange (β chain) and the peptide in green. (**B**) The model for A2-YLE with the peptide colored dark green. (**C**) The model for A2-YLE-3A with the peptide colored orange (for A2-YLE-3A, there were 2 molecules in the asymmetric unit, but these were virtually identical in terms of omit and density maps, so only copy 1 is shown here). (**D**) The model for A2-YLE-5A with the peptide colored pink. Reprinted with permission from reference^31^. Please click here to view a larger version of this figure.


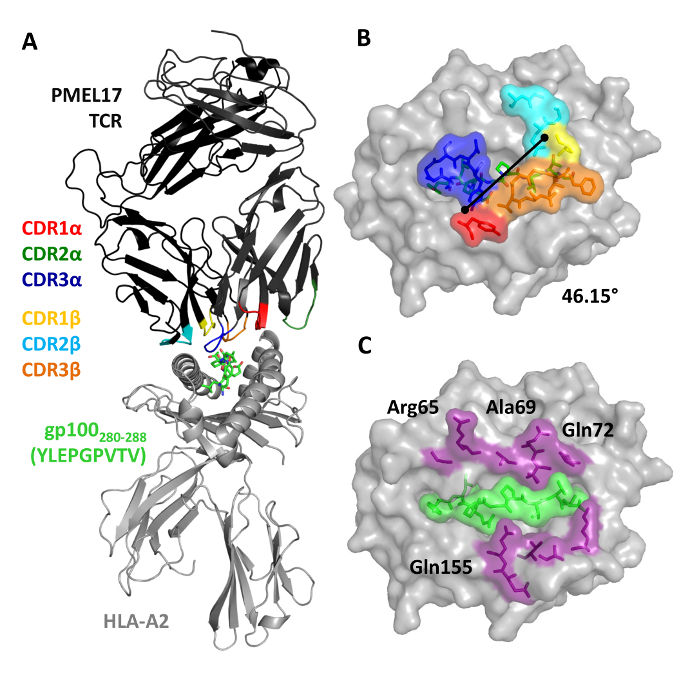
**Figure 2: Overview of the PMEL17 TCR in Complex with A2-YLE-9V. **(**A**) Cartoon representation of the PMEL17 TCR-A2-YLE-9V complex. The TCR is colored black; TCR CDR loops are shown (red, CDR1α; dark green, CDR2α; blue, CDR3α; yellow, CDR1β; aqua, CDR2β; orange, CDR3β); and the HLA-A*0201 is depicted in gray. The YLE-9V peptide is represented by green sticks. (**B**) Surface and stick representations of residues of the PMEL17 TCR CDR loops (color-coded as in A) that contact the A2-YLE surface (A2, gray; YLE-9V, green sticks). The black diagonal line indicates the crossing angle of the TCR with respect to the long axis of the YLEPGPVTV peptide (46.15°). (**C**) Contact footprint of the PMEL17 TCR on the A2-YLE-9V surface (A2, gray); purple and green (surface and sticks) indicate the HLA-A*0201 and YLE residues, respectively, contacted by the gp100 TCR. Cut-off of 3.4 Å for hydrogen bonds and 4 Å for van der Waals contacts. Reprinted with permission from Reference 31. Please click here to view a larger version of this figure.


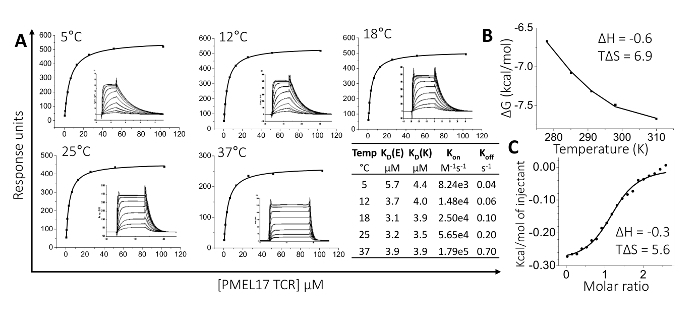
**Figure 3:Thermodynamic Analysis of the PMEL17 TCR-A2-YLE Interaction. **(**A**) PMEL17 TCR equilibrium-binding responses to A2-YLE at 5, 12, 18, 25, and 37 °C across nine to ten TCR serial dilutions. SPR raw and fitted data (assuming 1:1 Langmuir binding) are shown in the inset of each curve and were used to calculate K_on_ and K_off_ values using a global-fit algorithm (BIAevaluation 3.1). The table shows equilibrium-binding (K_D_ (E)) and kinetic-binding constants (K_D_ (K) = K_off_/K_on_) at each temperature. The equilibrium binding constant (K_D_, µM) values were calculated using a nonlinear fit (y = (P1x)/(P2+x)). (**B**) The thermodynamic parameters were calculated according to the Gibbs-Helmholtz equation (ΔG° = ΔH° − TΔS°). The binding free energies, ΔG° (ΔG° = -RTlnK_D_), were plotted against temperature (K) using a nonlinear regression to fit the three-parameter equation (y = ΔH°+ΔCp°*(x-298)-x*ΔS°-x*ΔCp°*ln(x/298)). Enthalpy (ΔH°) and entropy (TΔS°) at 298 K (25 °C) are shown in kcal/mol and were calculated by a non-linear regression of temperature (K) plotted against the free energy (ΔG°). (**C**) Isothermal calorimetric titration (ITC) measurements for the PMEL17 TCR-A2-YLE interaction. Enthalpy (ΔH°) and entropy (TΔS°) at 298 K (25 °C) are shown in kcal/mol. Reprinted with permission from reference^31^. Please click here to view a larger version of this figure.


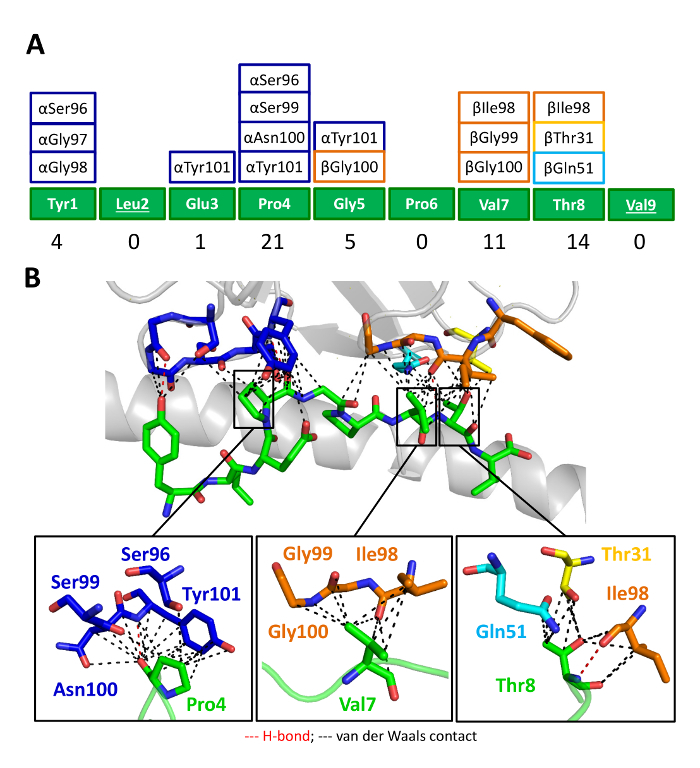
**Figure 4: The PMEL17 CDR Loops Focus on Peptide Residues Pro4, Val7, and Thr8. **(**A**) Schematic representation of the contacts between the YLE-9V peptide and the PMEL17 CDR loop residues (color-coded as in Figure 2A). The numbers at the bottom of the panel show the total contacts between the TCR and the peptide. (**B**) Contacts between the PMEL17 TCR and the YLE-9V peptide (green sticks) showing the van der Waals contacts (black dashed lines) and hydrogen bonds (red dashed lines) made by the TCR CDR3α (blue), CDR1β (yellow), CDR2β (aqua), and CDR3β (orange) loops. In the lower panel is a close view of the contacts between YLE Pro4, Val7, and Thr8, respectively, and TCR CDR loop residues (sticks color coded as in Figure 1A). Cut-off of 3.4 Å for hydrogen bonds and 4 Å for van der Waals contacts. Reprinted with permission from reference^31^. Please click here to view a larger version of this figure.


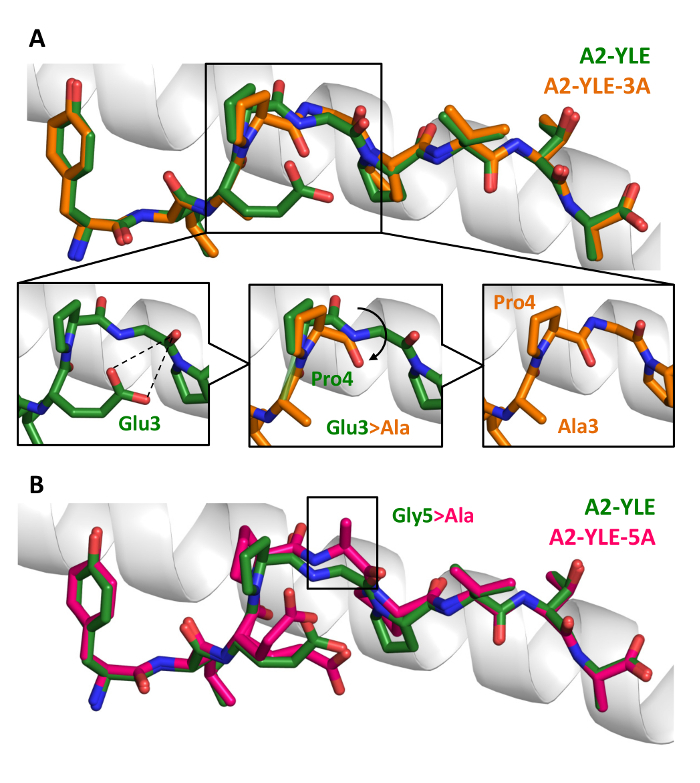
Figure 5: Conformational Comparison of YLE, YLE-3A, and A2-YLE-5A Peptides Presented by HLA-A*0201. (**A**) YLE (dark green sticks) and YLE-3A (orange sticks) peptide alignment by the superimposition of HLA-A*0201 α1 helix (gray cartoon). Boxed residues indicate the mutation of Glu3 into an alanine. The insets show how the Glu3Ala substitution causes a shift in position (black arrow) of neighbor residue Pro4 in the A2-YLE-3A structure compared to the A2-YLE structure. (**B**) YLE (dark green sticks) and YLE-5A (pink sticks) peptide alignment by the superimposition of HLA-A*0201 α1 helix (gray cartoon). The boxed residues indicate the mutation of glycine 5 into an alanine. Reprinted with permission from reference^31^. Please click here to view a larger version of this figure.

**Table:** 

**TCR**	**CDR1α**	**CDR2α**	**CDR3α**	**CDR1β**	**CDR1β**	**CDR1β**
**PMEL17**	DSAIYN	IQSSQRE	CAVLSSGGSNYKLTFG	SGHTA	FQGTGA	CASSFIGGTDTQYFG
**gp100**	TSINN	IRSNERE	CATDGDTPLVFG	LNHDA	SQIVND	CASSIGGPYEQYFG
**MPD**	KALYS	LLKGGEQ	CGTETNTGNQFYFG	SGHDY	FNNNVP	CASSLGRYNEQFFG
**296**	DSASNY	IRSNVGE	CAASTSGGTSYGKLTFG	MNHEY	SMNVEV	CASSLGSSYEQYFG

**Table 1: Alignment of TCR CDR3 Regions of PMEL17, gp100, MPD, and 296 gp100-specific TCRs. **Reprinted with permission from reference^31^.

**Table:** 

**Peptide sequence**	**Peptide**	**PMEL17 TCR***TRAV21 TRBV7-3* Affinity K_D_	**gp100 TCR***TRAV17 TRBV19* Affinity K_D_
YLEPGPVTA	YLE	7.6 ±2 μM	26.5 ±2.3 μM
YLEPGPVT**V**	YLE-9V	6.3 ±1.2 μM	21.9 ±2.4 μM
**A**LEPGPVTA	YLE-1A	15.9 ±4.1 μM	60.6 ±5.4 μM
YL**A**PGPVTA	YLE-3A	No binding	No binding
YLE**A**GPVTA	YLE-4A	19.7 ±1.3 μM	144.1 ±7.8 μM
YLEP**A**PVTA	YLE-5A	>1 mM	>1mM
YLEPG**A**VTA	YLE-6A	11.4 ±2.7 μM	954.9 ±97.8 μM
YLEPGP**A**TA	YLE-7A	31.1 ±4 μM	102.0 ±9.2 μM
YLEPGPV**A**A	YLE-8A	38.1 ±7.4 μM	121.0 ±7.5 μM

**Table 2: Affinity Analysis (K_D_) of PMEL17 TCR and gp100 TCR to gp100_280-288 _peptide variants. **Reprinted with permission from reference^31^.

**Table:** 

**Parameters**	**PMEL17 TCR-A2-YLE-9V**	**A2-YLE**	**A2-YLE-3A**	**A2-YLE-5A**
**PDB code**	**5EU6**	**5EU3**	**5EU4**	**5EU5**
**Dataset statistics**				
Space group	P1	P1 21 1	P1	P1 21 1
Unit cell parameters (Å)	a= 45.52, b= 54.41, c= 112.12, a=85.0°, b=81.6°, g=72.6°	a= 52.81, b= 80.37, c= 56.06, b=112.8°	a= 56.08, b= 57.63, c= 79.93, a=90.0°, b=89.8°, g=63.8°	a= 56.33, b= 79.64, c= 57.74, b=116.2°
Radiation source	DIAMOND I03	DIAMOND I03	DIAMOND I02	DIAMOND I02
Wavelength (Å)	0.9763	0.9999	0.9763	0.9763
Measured resolution range (Å)	51.87 – 2.02	45.25 – 1.97	43.39 – 2.12	43.42 – 1.54
Outer Resolution Shell (Å)	2.07 - 2.02	2.02 – 1.97	2.18 – 2.12	1.58 - 154
Reflection observed	128,191 (8,955)	99,442 (7,056)	99,386 (7,463)	244,577 (17,745)
Unique reflections	64,983 (4,785)	30,103 (2,249)	49,667 (3,636)	67,308 (4,962)
Completeness (%)	97.7 (96.7)	98.5 (99.3)	97.4 (96.7)	99.6 (99.9)
Multiplicity	2.0 (1.9)	3.3 (3.1)	2.0 (2.1)	3.6 (3.6)
I/Sigma(I)	5.5 (1.9)	7.2 (1.9)	6.7 (2.3)	13 (2.3)
Rpim (%)	5.7 (39.8)	8.8 (44.7)	8.7 (41.6)	4.5 (35.4)
R_merge_ (%)	7.8 (39.6)	9.8 (50.2)	8.7 (41.6)	5.0 (53.2)
**Refinement statistics**				
Resolution (Å)	2.02	1.97	2.12	1.54
No reflections used	61688	28557	47153	63875
No reflection in Rfree set	3294	1526	2514	3406
R_cryst_ (no cut-off) (%)	18.1	19.7	17.2	17.0
R_free_	22.2	25.5	21.1	20.1
**Root mean square deviation from ideal geometry**				
Bond lengths (Å)	0.018 (0.019)*	0.019 (0.019)*	0.021 (0.019)*	0.018 (0.019)*
Bond angles (°)	1.964 (1.939)*	1.961 (1.926)*	2.067 (1.927)*	1.914 (1.936)*
Overall coordinate error (Å)	0.122	0.153	0.147	.055
Ramachandran Statistics				
Most Favoured	791 (96%)	371 (98%)	749 (99%)	384 (98%)
Allowed	32 (4%)	6 (2%)	10 (1%)	5 (1%)
Outliers	2 (0%)	3 (1%)	1 (0%)	2 (0%)

**Table 3:Data Reduction and Refinement Statistics (molecular replacement).** Reprinted with permission from reference^31^. Values in parentheses are for the highest resolution shell.

**Table:** 

**HLA/peptide residue**	**TCR residue**	**No. vdW (≤4Å)**	**No. H-bonds (≤3.4Å)**
Gly62	αGly98	3	
	αSer99	1	
Arg65	αSer99	2	
Arg65 ^O^	αAsn100 ^Nδ2^	2	1
Arg65 ^NH1^	βAsp58 ^Oδ2^		1
	βSer59	8	
Lys66	αGly98	1	
	αSer99	4	
	αAsn100	4	
Ala69	αAsn100	2	
	βAla56	2	
Gln72 ^Nε2^	βGln51 ^O^	3	1
	βGly54	7	
	βAla55	1	
Thr73	βGln51	1	
Val76	βGln51	3	
	βGly52	2	
Lys146	βPhe97	3	
	βIle98	3	
Ala150	βIle98	1	
	βAsp102	3	
Val152	βIle98	1	
Glu154	αTyr32	1	
Gln155 ^N^	αTyr32 ^OH^	4	1
Gln155 ^Oε1^	βThr101 ^N^	10	1
Tyr1^OH^	αGly97 ^O^	1	1
	αGly98	1	
	αSer96	1	
Glu3	αTyr101	1	
Pro4	αSer96	1	
	αSer99	1	
	αAsn100	4	
Pro4 ^O^	αTyr101^N^	14	1
Gly5	αTyr101	3	
	βGly100	2	
Val7	βIle98	7	
	βGly99	2	
	βGly100	2	
Thr8	βThr31	5	
	βGln51	1	
	βPhe97	1	
Thr8 ^N^	βIle98 ^O^	6	1

**Table 4: PMEL17 TCR-A2-YLE-9V Contact Table.** Reprinted with permission from reference^31^.

## Discussion

The protocols outlined here provide a framework for the molecular and cellular dissection of T-cell responses in the context of any human disease. Although cancer was the main focus of this study, we have used very similar approaches to investigate T-cell responses to viruses^32^,^33^,^34^,^35^,^36^,^37^ and during autoimmunity^38^,^39^,^40^. Furthermore, we have used these techniques more broadly to understand the molecular principles that govern T-cell antigen recognition^2^,^19^,^41^,^42^. Indeed, the unpredictable nature of modifications to peptide residues, even those outside of the of the TCR contact residues, impacts T-cell recognition has important implications for the design of heteroclitic peptides. These findings have directly contributed to the development of novel T-cell therapies, including peptide vaccines^6^,^43^ and artificial high-affinity TCRs^3^,^4^,^5^,^20^,^44^, as well as of enhanced diagnostics^45^,^46^,^47^.

Critical steps within the protocol The generation of a highly pure, functional protein is essential for all of the methods outlined in this paper.

Modifications and troubleshooting Difficulties in generating highly pure protein often relate to the expression of highly-pure, insoluble IBs from the *E. coli* expression system. Usually, modifying the expression protocol (*e.g., *inducing at different optical densities, using different *E. coli* strains, or using different media formations) resolves these issues.

Limitations of the technique These techniques use soluble protein molecules (TCR and pHLA) that are normally expressed at the cell surface. Thus, it is important to ensure that structural/biophysical findings are consistent with cellular approaches to confirm biological significance.

Significance of the technique with respect to existing/alternative methods Through the use of X-ray crystallography and biophysics substantiated through functional analysis, we and others have demonstrated that TCRs specific for cancer epitopes are generally characterized by low binding affinities^48^. This low TCR affinity may help explain why T cells are not naturally effective at clearing cancer. High-resolution atomic structures of complexes between anti-cancer TCRs and cognate tumor antigens are starting to reveal the molecular basis for this weak affinity. Furthermore, these studies are helpful for determining the mechanisms that underlie the therapeutic interventions designed to overcome this issue, seeding future improvements^16^. In this study, we examined the first structure of a naturally-occurring αβTCR in complex with a gp100 HLA-A*0201-restricted melanoma epitope. The structure, combined with an in-depth biophysical examination, revealed the overall binding mode of the interaction. We also uncovered an unexpected molecular switch, which occurred in a mutated form of the peptide, that abrogated TCR binding (assessed using surface plasmon resonance) and CD8^+^ T-cell recognition (functional experiments). It was only possible to demonstrate this new mechanism of HLA antigen presentation using the high-resolution methods described.

Future applications or directions after mastering this technique Overall, our results demonstrate the power of X-ray crystallography and biophysical methods when combined with robust functional analyses. Using these approaches, it is possible to dissect out precise molecular mechanisms that govern T-cell antigen recognition. Indeed, it is also possible to use this approach to solve the structure of unligated TCRs, demonstrating how conformational changes can play a role during antigen discrimination^49^,^50^,^51^. A better understanding of the highly complex and dynamic nature that underpins TCR-pHLA interactions also has obvious implications for therapy design. Being able to directly "see" the molecules that are being therapeutically targeted, as well as the effect that modifications have on antigen recognition, will clearly improve the development of these medicines going forward. In this study, we show that even changes in a single peptide residue that is not heavily engaged by a TCR can unpredictably transmit structural changes to other residues in the HLA-bound peptide, which, in turn, dramatically alters T-cell recognition. A more complete understanding of the molecular mechanisms employed during T-cell antigen recognition will be hugely beneficial when designing future therapies for a wide range of human diseases.

## Disclosures

The authors have no conflicts of interest or competing financial interests. 
